# Feasibility and possible value of quantitative semi-automated diffusion weighted imaging volumetry of neuroblastic tumors

**DOI:** 10.1186/s40644-020-00366-3

**Published:** 2020-12-17

**Authors:** Sebastian Gassenmaier, Ilias Tsiflikas, Jörg Fuchs, Robert Grimm, Cristian Urla, Michael Esser, Simon Maennlin, Martin Ebinger, Steven W. Warmann, Jürgen F. Schäfer

**Affiliations:** 1 Department of Diagnostic and Interventional Radiology, University Hospital Tuebingen, Hoppe-Seyler-Straße 3, 72076 Tuebingen, Germany; 2 Department of Pediatric Surgery and Pediatric Urology, University Children’s Hospital Tuebingen, Tuebingen, Germany; 3 Siemens Healthcare GmbH, Erlangen, Germany; 4 Department of Pediatric Hematology and Oncology, University Children’s Hospital Tuebingen, Tuebingen, Germany

**Keywords:** Diagnostic imaging, Magnetic resonance imaging, Diffusion magnetic resonance imaging, Neuroblastoma

## Abstract

**Background:**

To assess the feasibility and possible value of semi-automated diffusion weighted imaging (DWI) volumetry of whole neuroblastic tumors with apparent diffusion coefficient (ADC) map evaluation after neoadjuvant chemotherapy.

**Methods:**

Pediatric patients who underwent surgical resection of neuroblastic tumors at our institution from 2013 to 2019 and who received a preoperative MRI scan with DWI after chemotherapy were included. Tumor volume was assessed with a semi-automated approach in DWI using a dedicated software prototype. Quantitative ADC values were calculated automatically of the total tumor volume after manual exclusion of necrosis. Manual segmentation in T1 weighted and T2 weighted sequences was used as reference standard for tumor volume comparison. The Student’s t test was used for parametric data while the Wilcoxon rank sum test and the Kruskal-Wallis test were applied for non-parametric data.

**Results:**

Twenty seven patients with 28 lesions (neuroblastoma (NB): *n* = 19, ganglioneuroblastoma (GNB): *n* = 7, ganglioneuroma (GN): *n* = 2) could be evaluated. Mean patient age was 4.5 ± 3.2 years. Median volume of standard volumetry (T1w or T2w) was 50.2 ml (interquartile range (IQR): 91.9 ml) vs. 45.1 ml (IQR: 98.4 ml) of DWI (*p* = 0.145). Mean ADC values (× 10^− 6^ mm^2^/s) of the total tumor volume (without necrosis) were 1187 ± 301 in NB vs. 1552 ± 114 in GNB/GN (*p* = 0.037). The 5th percentile of ADC values of NB (614 ± 275) and GNB/GN (1053 ± 362) provided the most significant difference (*p* = 0.007) with an area under the curve of 0.848 (*p* < 0.001).

**Conclusions:**

Quantitative semi-automated DWI volumetry is feasible in neuroblastic tumors with integrated analysis of tissue characteristics by providing automatically calculated ADC values of the whole tumor as well as an ADC heatmap. The 5th percentile of the ADC values of the whole tumor volume proved to be the most significant parameter for differentiation of the histopathological subtypes in our patient cohort and further investigation seems to be worthwhile.

**Supplementary Information:**

The online version contains supplementary material available at 10.1186/s40644-020-00366-3.

## Background

Neuroblastoma (NB) is the most common extracranial pediatric cancer with a total incidence of 10.2 cases per million children under 15 years of age [1, 2]. In the diagnostic pathway of NB and the rather benign groups of ganglioneuroblastoma (GNB) and ganglioneuroma (GN) radiological imaging features a key role [3]. Imaging findings are not only used for risk stratification via the image defined risk factors according to the current International Neuroblastoma Risk Group (INRG) staging system, but also to differentiate between other tumor entities [3–7]. During follow-up, imaging is further needed for evaluation of response after chemotherapy and for detection of tumor relapse. Most common imaging modalities in pediatric patients with neuroblastic tumors involve ultrasound (US), computed tomography (CT), magnetic resonance imaging (MRI), and ^123^Iodine-meta-iodobenzylguanidine (^123^I-MIBG) scintigraphy [3, 8]. However, it is still under debate which imaging modality provides the best results regarding prognosis and control of treatment response [9–12]. Moreover, high levels of ionizing radiation caused by diagnostic imaging during follow-up period should not be neglected [13, 14]. In this regard, MRI has become easier to use as standard imaging procedure due to the technical advances [15]. Recent studies have shown that especially diffusion weighted imaging (DWI) may have an impact on diagnosis and prediction of prognosis [4, 16–19]. Whereas Aslan et al. demonstrated the feasibility of DWI for the differentiation between nephroblastoma and NB, it has also been shown that apparent diffusion coefficient (ADC) maps can be used for tissue characterization to distinguish between NB, GNB, and GN [4, 16, 20]. Additionally, the changes of ADC values during chemotherapy can be used as a prognostic factor for event free survival [17].

Usually, regions of interests (ROI) or single slices have been selected for quantification of ADC values [4, 16, 17]. However, single ROI or slices do not reflect the exact tissue component of the characteristically inhomogeneous NB, especially after neoadjuvant therapy with increasing variability of tumor masses. Therefore, our study aimed to assess the feasibility and diagnostic value of semi-automated DWI volumetry of whole neuroblastic tumors with automated ADC analysis before and after neoadjuvant chemotherapy.

## Methods

### Study population

This retrospective, monocentric study was approved by the institutional review board with waiver of informed consent. Forty-five patients, who underwent gross total resection (GTR) of neuroblastic tumors (NB, GNB, and GN) after neoadjuvant therapy at our institution between 2013 and 2019, were included. Additionally, the availability of a preoperative MRI scan within 100 days before operation including sufficient DWI with at least two different b-values and ADC maps was mandatory. As many patients were referred for surgical resection only, no uniform imaging protocol was available, and 11 patients had to be excluded due to missing DWI. Seven patients were excluded due to artifacts in DWI. Therefore, the final study group consisted of 27 patients.

In cases with available MRI scans of the initial tumor mass before chemotherapy, we performed an additional ADC analysis of the whole tumor volume prior to chemotherapy for comparison and evaluation of therapy response.

The final histological diagnosis was obtained from the histopathology report of the resected specimen. Risk group stratification was performed according to the current guidelines and patients were assigned to three groups: low risk (LR), intermediate risk (IR), and high risk (HR) [21].

### Imaging protocol and tumor volumetry

No uniform imaging protocol was available as mentioned above. Repetition time (TR) ranged from 3.4–457 ms and echo time (TE) from 1.1–9.5 ms for T1 weighted imaging. For T2 weighted imaging, a TR of 1800 ms – 6900 ms and a TE of 69–146 ms was applied. DWI was acquired using epi planar imaging with b-values set of 0 and 1000 s/mm^2^ and sets of 50, 400 and 800 s/mm^2^. TR ranged from 2500 ms – 12,500 ms as well as TE from 52 ms – 79 ms. All tumors were segmented by one radiologist with 4 years of experience in image post- processing procedures after the boundaries were determined in consensus with a board-certified pediatric radiologist with 28 years of experience. Both readers were blinded to the histopathological report. At first manual tumor segmentation in axial T2 weighted sequences was performed by delineating the tumor margins in all slices using standard postprocessing software (syngo.via, Siemens Healthineers, Erlangen; Germany). If T2 weighted sequences were not available or blurred by artifacts, a postcontrast T1 weighted Dixon volumetric interpolated breath-hold (VIBE) sequence was used instead. The volumetry in these standard sequences was used as reference for comparison. After an interval of 4 weeks, DWI derived volume and quantitative image analysis was semi-automatically calculated using a dedicated software prototype (MR Total Tumor Load, Siemens Healthineers, Erlangen; Germany). For the analysis of inter- and intra-observer variability, a subset of 5 patients were segmented by 5 radiologists (experience ranging from one to 28 years) twice with a gap of at least 4 weeks with the software prototype. To depict all facets of the study group, this subset consisted of two NB, two GNB and one GN. Tumor location was in two cases thoracic and in three cases abdominal. Segmentation time was recorded.

### Quantitative semi-automated imaging analysis tool

In essence, the software uses the acquired diffusion weighted images to calculate a synthetic diffusion weighted image at b = 1000 s/mm^2^ and creates an initial 3D ADC tumor mask by threshold-based segmentation on the DWI signal intensity. Although any b-values can be calculated with this software, in this study all analyses were performed using a b-value of 1000 s/mm^2^.This ADC mask could be further edited manually in axial, coronal and sagittal planes in single slices or in maximum intensity projections to remove undesired structures beyond the area of interest such as the brain, the spleen, and the bladder. Additionally, it was possible to add semi-automatically false negative areas (tumor tissue that was not included in the segmentation) with a brush tool. Based on this mask, the lesion volume and ADC histogram parameters were calculated including mean and median values as well as the 5th percentile. Voxels with an ADC value (× 10^− 6^ mm^2^/s) of less than 750 were superimposed in red on the DWI maximum intensity projection, in yellow for the ADC range of 750–1500 and in green for voxels with an ADC value exceeding 1500. The ADC mask could also be displayed in three orthogonal planes.

### Total tumor ADC calculation

After calculation of the whole DWI derived volume, necrosis in all tumors was excluded manually for the calculation of the ADC values. For this process all cases were assessed in consensus by the two radiologists mentioned above. All image slices were analyzed in DWI with a calculated b-value of 1000 s/mm^2^, the corresponding ADC map, as well as pre- and postcontrast T1 weighted imaging. All diffusion restricted areas were correlated visually and matched in T1 weighted imaging. In case of hyperintense signal in precontrast imaging and no enhancement in postcontrast imaging, these areas were defined as necrotic. Following this analysis, all necrotic areas were delineated manually on every slice of the ADC map and excluded for ADC analysis of the semi-automated segmented tumor tissue. Additionally, to compare whole tumor ADC analysis with an evaluation of a single slice, a ROI was drawn in a mid axial slice (avoiding necrosis).

### Statistical analysis

Statistical analysis was performed using JMP14 (SAS Institute, Cary, North Carolina; USA) and MedCalc version 18.1 (MedCalc Software, Ostend; Belgium). The Shapiro-Wilk test was used to test for normal distribution. Continuous variables were displayed as mean ± standard deviation. The Student’s t test was used for parametric data while the Wilcoxon rank sum test and the Kruskal-Wallis test were applied for non-parametric data. Pearson’s correlation coefficient was used to determine the relationship between DWI and T1/2 weighted volume. Due to the low sample size of patients with imaging prior to chemotherapy, only non-parametric tests were used for this subanalysis. For the comparison of pre- and post-chemotherapy results of this subset respective tests for paired data were applied. Intraclass correlation coefficient (ICC) was used to determine inter- and intra-reader variability of the segmentation process.

Receiver operator characteristics (ROC) was performed to determine the diagnostic accuracy of ADC values for distinguishing the histological subtypes. The significance level alpha was set at 0.05.

## Results

### Patients` characteristics

Tumor volumetry and ADC analysis was successfully performed in 28 lesions of 27 included patients (final histopathological result after resection: NB: *n* = 19; GNB: *n* = 7; GN: *n* = 2). The biopsies of both GN resulted prior to chemotherapy in the diagnosis of GNB and underwent therefore neoadjuvant chemotherapy.

Mean patient age at preoperative MRI imaging was 4.5 ± 3.2 years. Seventeen patients were male (63%). MRI was performed 18 ± 24 days before operation (range 1–91 days).

Further characteristics are displayed in Table 1.

**Table 1:** Characteristics of the study group

Characteristics	Values
Patients	*n* = 27 with 28 lesions
*Age*	
Mean age ± std.	4.5 ± 3.2 years
Range	0.3–11.5 years
*Diagnosis*	
Neuroblastoma	*n* = 18 (one bilateral resulting in 19 NB lesions)
Ganglioneuroblastoma	n = 7
Ganglioneuroma	n = 2
*Tumor localization*^a^	
Cervical	n = 1
Thoracic	*n* = 4^a^
Abdominal (extraadrenal)	*n* = 16^a^
Adrenal	*n* = 7^b^
Pelvic	n = 1
*Genetic alterations*	
*MYCN* amplification	*n* = 6
Positive 1p-deletion	n = 4
Imbalanced 1p-status	n = 2
^*123*^*I-MIBG scintigraphy findings*	
^123^I-MIBG: positive	*n* = 11
^123^I-MIBG: negative	n = 1
^123^I-MIBG: unclear	n = 1
*Risk group stratification*	
High risk	*n* = 14
Intermediate risk	n = 7
Low risk	n = 6

^a^One case of thoracic-abdominal manifestation

^b^One bilateral manifestation

*Abbreviations:* ^*123*^*I-MIBG* ^123^Iodine-meta-iodobenzylguanidine

### Risk factor analysis

Six patients were diagnosed with amplified *MYCN* gene status. In four patients, 1p deletion was observed and in two patients an imbalanced status. ^123^I-MIBG scintigraphy was performed in 13 patients with positive findings in 11 cases, negative findings in one case and unclear findings in one case. Risk group stratification resulted in 14 HR, seven IR, and six LR patients (Table 1).

### DWI volumetry versus standard volumetry and ADC analysis

In three cases a T1 weighted VIBE Dixon sequence was used instead of T2 weighted imaging for standard volumetry. The mean standard volume was 110.5 ± 218.7 ml versus 116.4 ± 230.8 ml for DWI volumetry (Table 2). The mean difference between DWI and standard volumetry was 5.9 ± 18.1 ml. The absolute difference was 9.5 ± 14.2 ml. There was no significant difference between DWI and standard volumetry (*p* = 0.145). Bland-Altman analysis is displayed in Additional file 1. Pearson correlation was perfect (0.999) between DWI and standard volumetry (*p* < 0.001). Regarding the different histological subgroups there was no significant difference of tumor volumes between NB (105.5 ± 257.3 ml), GNB (106.1 ± 103.0 ml), and GN (173.0 ± 166.8) using standard volumetry (*p* = 0.285) and also no significant difference using DWI (*p* = 0.270).

**Table 2:** Volumetric comparison of standard volumetry and semi-automated DWI volumetry after chemotherapy (*n* = 28 lesions)

	Standard volumetry	DWI volumetry	*p*-value
Overall	110.5 ± 218.7 ml	116.4 ± 230.8 ml	0.145
NB	105.5 ± 257.3 ml	112.6 ± 272.3 ml	0.073
GNB/GN	121.0 ± 110.9 ml	124.3 ± 113.0 ml	0.551
Low risk group	70.1 ± 100.8 ml	69.0 ± 96.6 ml	0.770
Intermediate risk group	88.8 ± 134.9 ml	97.9 ± 143.4 ml	0.154
High risk group	141.5 ± 290.0 ml	149.3 ± 307.0 ml	0.146

Abbreviations: *DWI* Diffusion weighted imaging, *NB* Neuroblastoma, *GNB* Ganglioneuroblastoma, *GN* Ganglioneuroma

ICC for inter-reader variability of DWI volumetry was 0.972. ICC for intra-reader variability ranged from 0.975–0.999. Segmentation time resulted in 402 ± 211 s.

**Fig. 1:**
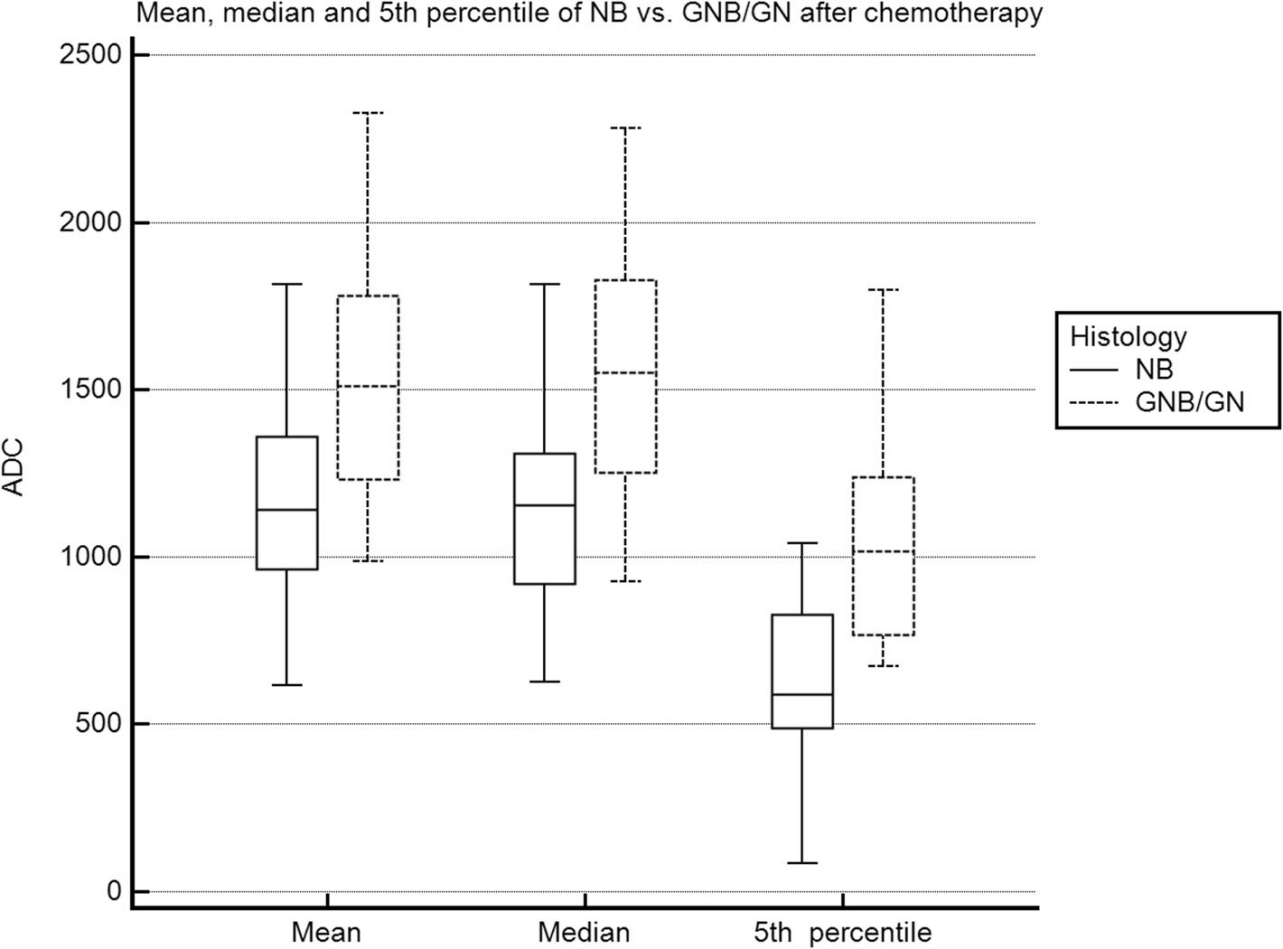
ADC value histogram of NB and GNB/GN. Figure 1 shows the mean, median, and 5th percentile of the total tumor ADC values (× 10^− 6^ mm^2^/s) of NB and GNB/GN, respectively. *Abbreviations: NB: neuroblastoma; GNB: ganglioneuroblastoma; GN: ganglioneuroma*

Due to the low sample size of GN, we combined GNB and GN to one group as performed in previously published studies for ADC comparison (Table 3) [17]. Mean total tumor ADC values after neoadjuvant chemotherapy were significantly different in NB (1187 ± 301 × 10^− 6^ mm^2^/s) versus GNB/GN (1552 ± 114 × 10^− 6^ mm^2^/s; *p* = 0.037). Median values were also significantly different between NB and GNB/GN with a median of 1163 ± 317 × 10^− 6^ mm^2^/s vs. 1560 ± 417 × 10^− 6^ mm^2^/s (*p* = 0.026). The biggest difference between NB and GNB/GN was observed analyzing the 5th percentile of ADC values (NB: 614 ± 275 × 10^− 6^ mm^2^/s; GNB/GN: 1053 ± 362 × 10^− 6^ mm^2^/s; *p* = 0.007; Fig. 1). There was no significant difference between the three risk groups (HR, IR, LR) of NB and GNB/GN regarding ADC values (Table 4).

**Table 3:** Total tumor ADC value (× 10^− 6^ mm^2^/s) analysis of NB and GNB/GN prior to chemotherapy (*n* = 10 lesions) and post-chemotherapy (*n* = 28 lesions)

	NB	GNB/GN	*p*-value
Mean ADC prior to chemo	964 ± 115	1578 ± 377	0.028
Mean ADC post-chemo	1187 ± 301	1552 ± 114	0.037
Median ADC prior to chemo	893 ± 138	1590 ± 394	0.028
Median ADC post-chemo	1163 ± 317	1560 ± 417	0.026
5th percentile prior to chemo	548 ± 103	985 ± 280	0.016
5th percentile post-chemo	614 ± 275	1053 ± 362	0.007

Abbreviations: *ADC* Apparent diffusion coefficient, *NB* Neuroblastoma, *GNB* Ganglioneuroblastoma, *GN* Ganglioneuroma

**Table 4:** ADC values (× 10^− 6^ mm^2^/s) analysis in risk groups after chemotherapy (n = 28 lesions)

	Low Risk	Intermediate Risk	High Risk	*p*-value
Mean ADC	1298 ± 417	1346 ± 410	1287 ± 369	0.774
Median ADC	1282 ± 449	1366 ± 419	1258 ± 376	0.713
5th percentile	739 ± 344	686 ± 450	798 ± 350	0.864

*Abbreviations: ADC* Apparent diffusion coefficient

There was no significant difference between mean, median, and 5th percentile ADC values of a single axial slice compared to total tumor volume in the histopathological subgroups. However, overall (NB, GNB, and GN combined), the 5th percentile of the total tumor volume was significantly smaller compared to a single slice (755 ± 364 × 10^− 6^ mm^2^/s vs. 913 ± 483 × 10^− 6^ mm^2^/s; *p* = 0.009).

**Fig. 2:**
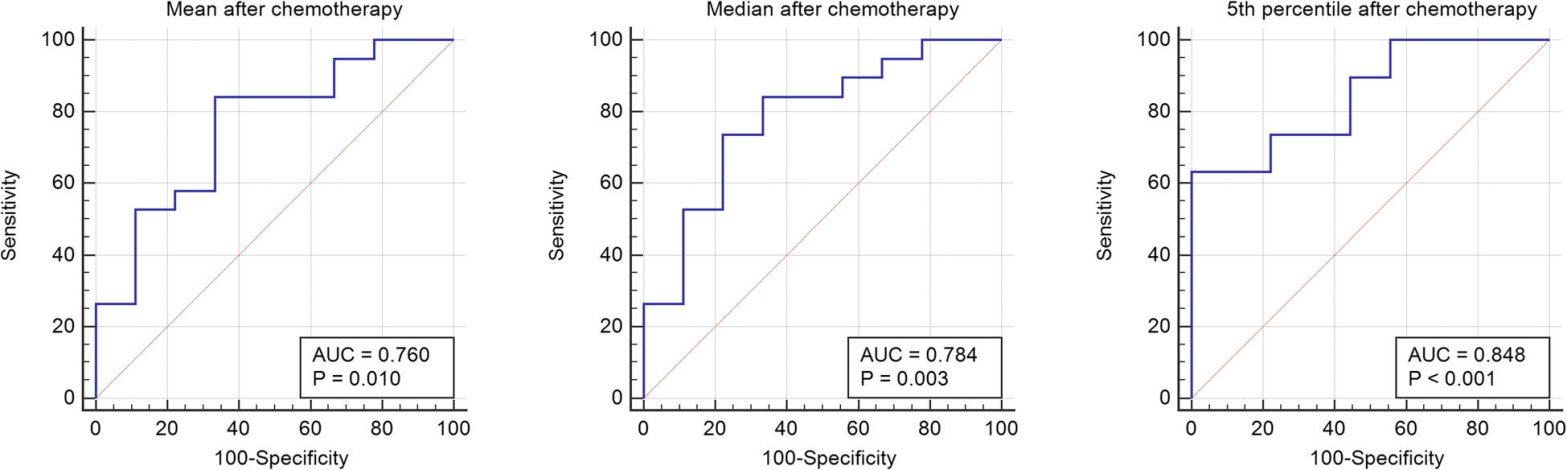
Receiver operator characteristics of NB and GNB/GN for mean, median and 5th percentile of the ADC values. Figure 2 shows receiver operator characteristics for mean, median, and 5th percentile of the ADC values (× 10^− 6^ mm^2^/s) of the whole tumor volume for differentiation between NB and GNB/GN after chemotherapy. The 5th percentile is suited best for distinguishing histopathological subtypes with an area under the curve of 0.848 after chemotherapy. *Abbreviations: ADC: apparent diffusion coefficient; NB: neuroblastoma; GNB: ganglioneuroblastoma; GN: ganglioneuroma*

ROC analysis revealed the 5th percentile of the total tumor volume as best predictor to distinguish between NB and GNB/GN. Using a value of 639 × 10^− 6^ mm^2^/s for the 5th percentile, this marker displayed a sensitivity of 63.2% and a specificity of 100% with an area under the curve (AUC) of 0.848 for distinguishing between NB and GNB/GN (*p* < 0.001). Applying the mean or median ADC value for differentiation of NB and GNB/GN showed no better performance in comparison to the 5th percentile with a sensitivity of 84.2% and a specificity of 66.7% for a mean of 1406 × 10^− 6^ mm^2^/s (AUC = 0.760; *p* = 0.010) and a sensitivity of 73.7% and a specificity of 77.8% for a median of 1269 × 10^− 6^ mm^2^/s (AUC = 0.784; *p* = 0.003), respectively (Fig. 2).

### Pre- and post-chemotherapy analysis

MRI with DWI was available before initial chemotherapy in ten patients (NB: *n* = 5; GNB: *n* = 4; GN: *n* = 1). Mean time between the first MRI and the preoperative MRI was 135 ± 68 days (range: 49–288). During chemotherapy all tumors except the ganglioneuroma showed response to treatment regarding size and volume. Using DWI for volumetry the mean difference between pre- and post-chemotherapy volume was 345.8 ± 359.9 ml (range: 94.3–1036.0 ml) in NB and 23.5 ± 26.4 ml (range: 0.6–66.5 ml) in GNB. The one GN in the study group increased its volume by 46.0 ml.

Initial mean ADC values were 964 ± 115 × 10^− 6^ mm^2^/s in NB versus 1578 ± 377 × 10^− 6^ mm^2^/s in GNB/GN (*p* = 0.028). Regarding median ADC values (893 ± 138 × 10^− 6^ mm^2^/s vs. 1590 ± 394 × 10^− 6^ mm^2^/s), there was also a significant difference (*p* = 0.028). The 5th percentile displayed also a significant difference between NB and GNB/GN with 548 ± 103 × 10^− 6^ mm^2^/s (NB) versus 985 ± 280 × 10^− 6^ mm^2^/s (GNB/GN) (*p* = 0.016) (Table 3). During chemotherapy mean ADC values of NB changed from 964 ± 115 × 10^− 6^ mm^2^/s to 1278 ± 280 × 10^− 6^ mm^2^/s, median ADC values changed from 893 ± 138 × 10^− 6^ mm^2^/s to 1251 ± 341 × 10^− 6^ mm^2^/s, and the 5th percentile changed from 548 ± 103 × 10^− 6^ mm^2^/s to 700 ± 211 × 10^− 6^ mm^2^/s. Mean ADC values of GNB/GN changed from 1578 ± 377 × 10^− 6^ mm^2^/s to 1501 ± 160 × 10^− 6^ mm^2^/s, median ADC values changed from 1590 ± 394 × 10^− 6^ mm^2^/s to 1536 ± 178 × 10^− 6^ mm^2^/s, and the 5th percentile changed from 985 ± 280 × 10^− 6^ mm^2^/s to 991 ± 177 × 10^− 6^ mm^2^/s. The evaluation within the histopathological subgroups before and after chemotherapy provided no significant difference within NB (*p* = 0.188 (mean); *p* = 0.188 (median); *p* = 0.313 (5th percentile)) and within GNB/GN (*p* = 0.625 (mean); *p* = 0.813 (median); *p* = 0.813 (5th percentile)).

Initial ADC values of the 5th percentile provided a sensitivity of 80% and a specificity of 100% for differentiating between NB and GNB/GN with a cut-off value of 616 × 10^− 6^ mm^2^/s (AUC = 0.960; *p* < 0.001). Using mean values, the cut-off was 1078 × 10^− 6^ mm^2^/s with a sensitivity of 100% and a specificity of 80% (AUC = 0.920; *p* < 0.001). Regarding median values, the cut-off was 1048 × 10^− 6^ mm^2^/s with a sensitivity of 100% and a specificity of 80% (AUC = 0.920; *p* < 0.001).

## Discussion

Our results show that semi-automated DWI derived tumor volume is comparable to manual standard segmentation methods without significant differences between these two methods in our study cohort. Moreover, our study indicates that whole tumor ADC analysis is feasible and might be applied for distinguishing between different histopathological types of neuroblastic tumors before and after chemotherapy. Hereby, the 5th percentile of ADC values proved to be the best marker for differentiation in our study cohort.

**Fig. 3:**
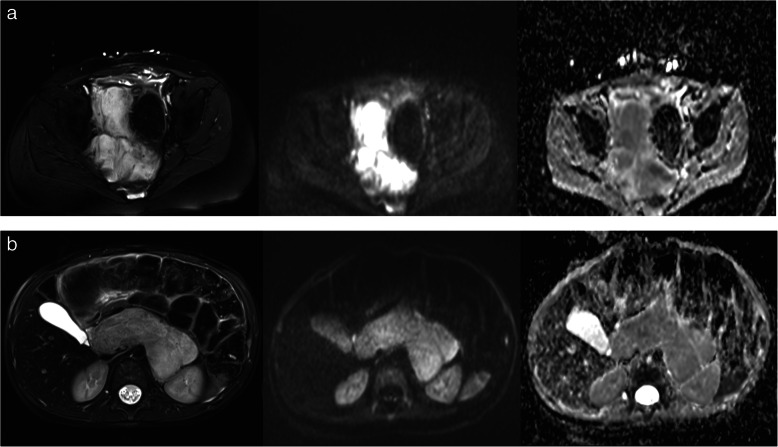
**a** and **b** Example of NB (**a**) and GNB (**b**) after neoadjuvant therapy in T2 weighted and diffusion weighted imaging. These figures depict an example of a NB (**a**) and GNB (**b**) after neoadjuvant therapy with T2 weighted and diffusion weighted imaging (b = 1000 s/mm^2^) as well as the corresponding ADC map. The NB appears more heterogenous than the GNB, especially in the ADC map. The 5th percentile of the ADC values resulted in 977.5 × 10^− 6^ mm^2^/s for the NB and 1199.0 × 10^− 6^ mm^2^/s for the GNB. *Abbreviations: NB: neuroblastoma; GNB: ganglioneuroblastoma; ADC: apparent diffusion coefficient*

**Fig. 4:**
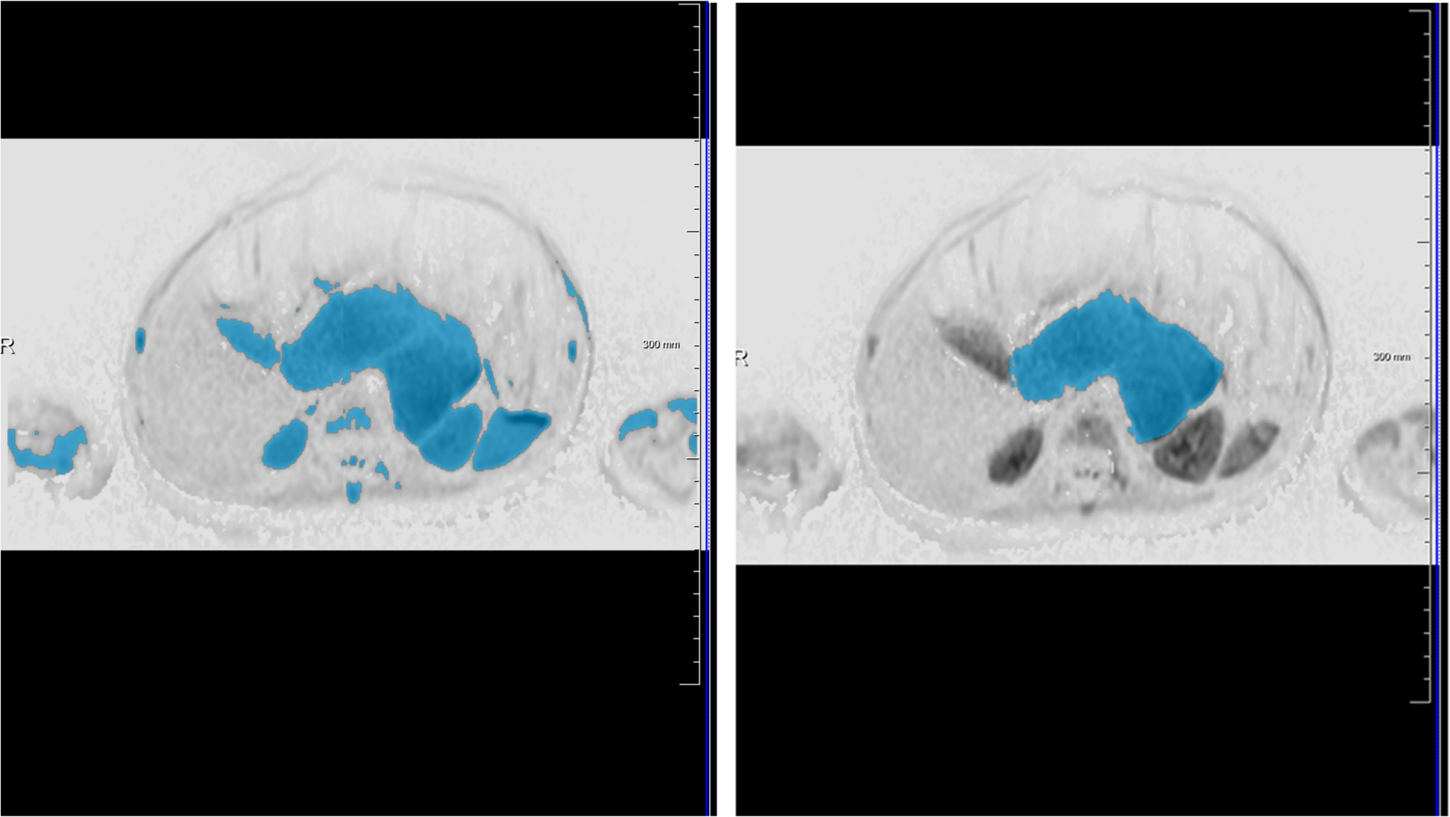
Screenshot of the software prototype. This figure shows a screenshot of the software prototype. This case displays the GNB of Fig. 3 b. After automated selection of diffusion restricted areas (left image) undesired structures were removed (right image). Diffusion restricted areas are marked in blue. *Abbreviations: GNB: ganglioneuroblastoma*

**Fig. 5:**
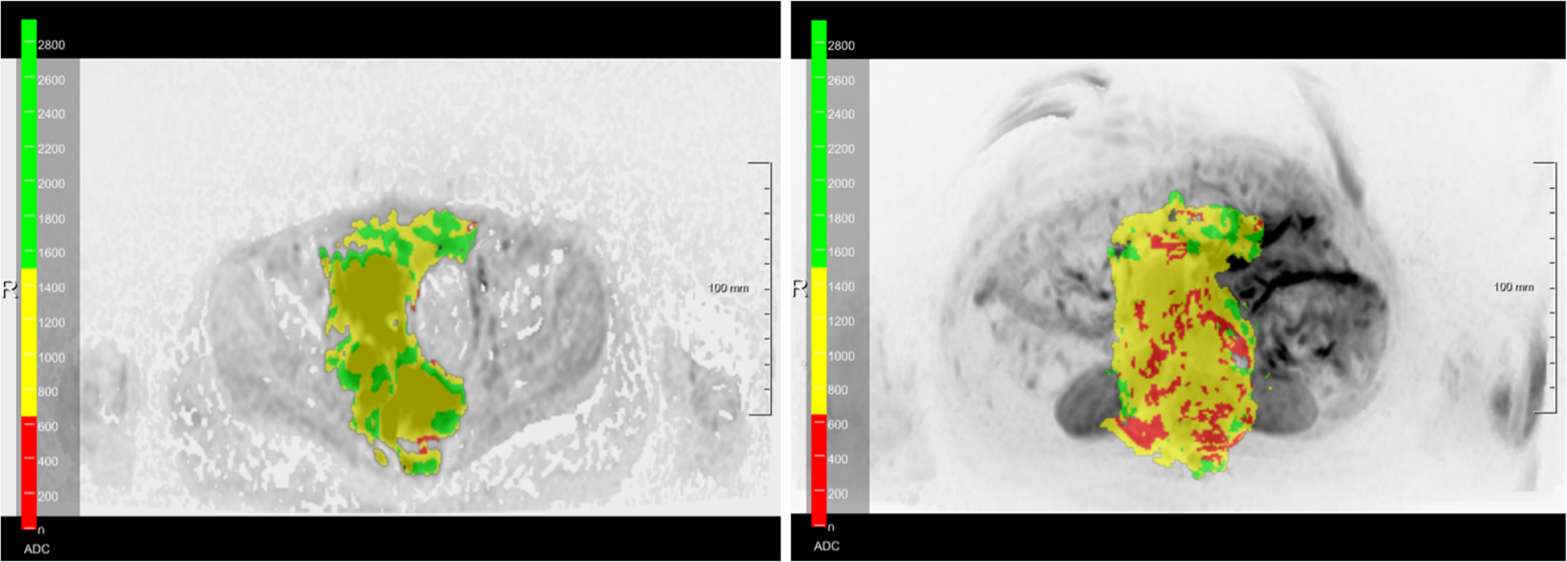
ADC heatmap of a neuroblastoma (Fig. 3a). This figure shows the distribution of the ADC values in a single slice of the NB of Fig. 3a as well as the corresponding maximum intensity projection in axial plane. Values below 750 × 10^− 6^ mm^2^/s are superimposed in red, values between 750 and 1500 × 10^− 6^ mm^2^/s in yellow, and values above 1500 × 10^− 6^ mm^2^/s in green. *Abbreviations: ADC: apparent diffusion coefficient; NB: neuroblastoma*

**Fig. 6:**
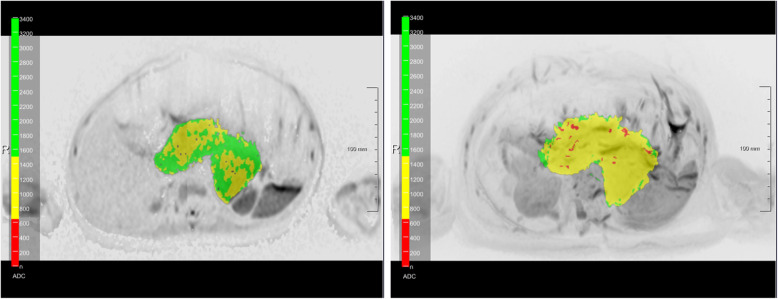
ADC heatmap of a ganglioneuroblastoma (Fig. 3 b). This figure shows the distribution of the ADC values in a single slice of the GNB of Fig. 3 b as well as the corresponding maximum intensity projection in axial plane. Values below 750 × 10^− 6^ mm^2^/s are superimposed in red, values between 750 and 1500 × 10^− 6^ mm^2^/s in yellow, and values above 1500 × 10^− 6^ mm^2^/s in green. *Abbreviations: ADC: apparent diffusion coefficient; GNB: ganglioneuroblastoma*

Our study results are in line with previously published results, which could show that ADC values differ between NB, GNB, and GN [16–18, 20]. However, in these mentioned studies a small ROI and large ROI approach were used, respectively. For example, Peschmann et al. described a mean ADC value of 760 ± 110 × 10^− 6^ mm^2^/s for NB and 1470 ± 230 × 10^− 6^ mm^2^/s for GNB/GN before chemotherapy [17]. In our study we observed mean values of 964 ± 138 × 10^− 6^ mm^2^/s for NB and 1578 ± 377 × 10^− 6^ mm^2^/s for GNB/GN prior to chemotherapy. This suggests that the use of a whole tumor segmentation approach provides slightly higher mean values by representing the tumor heterogeneity more accurately than using a single ROI only (Fig. 3a and b). Therefore, the 5th percentile seems to be a capable marker to differentiate between neuroblastic tumor types in whole tumor analysis. A disadvantage of single ROI measurements lies in the inadvertent misplacing of a ROI in an area with extraordinarily high or low ADC values compared to the rest of the tumor tissue leading to false conclusions regarding its characteristics. This risk might be diminished by analyzing the ADC values of the whole tumor as the tumor heterogeneity is better depicted. Additionally, as a good agreement between the readers was proven, semi-automated whole tumor analysis might be less prone to inter-reader variability compared to single ROI approach. However, although tumor heterogeneity might display an important issue, there was no significant difference between mean and median ADC values of single ROI compared to total tumor ADC analysis. This might be related to the small sample size. Therefore, this work displays primarily a feasibility study proving that the described approach can be applied in neuroblastic tumors. However, due to the rareness of this disease, further ideally multi-center studies are necessary to investigate the clinical impact and reliability of this technique. Despite these mentioned drawbacks, we think it can be assumed that whole tumor volume analysis displays the ground truth of ADC values more accurately than single slice analysis. However, due to the similarity of the values and time efficiency, especially the 5th percentile seems to be an interesting parameter that might also be useful in single ROI assessment. An example of the analysis via the software is displayed in Figs. 4, 5, and 6.

Regarding the clinical value of the proposed method it is of interest that the surgical approach in NB has been controversially discussed in the literature, especially in the presence of IDRF [3, 5]. It has previously been demonstrated that in intensively treated high risk NB patients, surgical removal of the tumor does not affect local recurrence or overall survival [22]. This contradicts the results from La Quaglia et al. who could show that GTR is correlated with improvement of overall survival in high risk NB patients [23]. Therefore, an additional benefit of providing all ADC values within the tumor mass displays the depiction in a 3D model that could be used as an intraoperative navigation system to remove at least the very aggressive parts of the tumor with low ADC values. Furthermore, this heat map could be applied in the execution of biopsies to prevent sampling errors, particularly if biopsies are being carried out minimally-invasively (Figs. 5 and 6).

The increase of ADC values during chemotherapy as well as tumor volume decrease is also similar with the results of previous studies [16, 17]. The analysis of ADC values of the whole tumor can be used for tumor response assessment. By providing a heat map of the ADC values prior to chemotherapy and after chemotherapy, tumor response might be better depicted, e. g. via subtraction methods. This might also be relevant for local radiation therapy to increase radiation dose in areas with low response and decrease dosage in areas with good response. Another upside might display the possibility of semi-automated response assessment with further technical development.

This study emphasizes the multiple advantages of MRI in neuroblastic tumors. For morphological imaging and assessment of image defined risk factors CT or MRI can be used. However, the current guidelines do not handle whole body MRI or at least MRI of the tumor region including DWI as standard of care, whereas an MIBG scan is routinely performed in all patients with neuroblastic tumors [8, 24]. It has previously been shown that MRI including DWI and ADC maps provide higher sensitivity of lesion detection with the compromise of worse specificity in comparison to MIBG [24–26]. Due to the higher spatial resolution and the advantages for soft tissue imaging and spinal involvement, MRI displays many advantages for staging and follow-up examinations [12]. Additionally, MRI including DWI can act as a one-stop-shop by providing morphological imaging, functional assessment, and tissue characterization of the mass within one examination. Another benefit in comparison to CT or MIBG scintigraphy is of course the radiation free imaging process. Owens et al. could show in a retrospective analysis that the mean radiation exposure in neuroblastic tumor patients that will experience a relapse is up to 125.2 millisievert prior to the relapse (64% of dose through CT and 18.2% through MIBG studies) [14]. These exposure levels could be drastically reduced by using MRI as standard of care for initial diagnosis and follow-up examinations although the need for anesthesia and sedation in very young patients must not be neglected.

### Limitations

Although the number of subjects is relatively small, it still represents one of the largest published cohorts analyzing ADC values of neuroblastic tumors due to the low incidence of this disease. Nevertheless, this investigation displays primarily a feasibility study investigating the methodology of the presented approach. Another issue that merits consideration is the absence of a uniform imaging protocol as not all imaging studies were performed at one institution. However, for DWI and ADC assessment a b-value of 1000 s/mm^2^ was used in all studies. Due to the heterogeneous texture and appearance of neuroblastic tumors the definition of tumor margins and exclusion of necrosis was assessed in consensus reading. However, the subset analysis showed an excellent agreement between the five readers. Although, no significant difference was found regarding the volumetry between standard volumetry and semi-automated DWI volumetry, this is not a proof for equivalent measuring methods. Further multicenter prospective studies with uniform imaging protocol are mandatory to verify our initial results on a lager study cohort.

## Conclusions

Quantitative semi-automated DWI volumetry is feasible in neuroblastic tumors with reliable tumor volume assessment. Additionally, by providing automatically calculated ADC values of the whole tumor it features a further analysis of the tissue characteristics that can be used for differentiation between histopathological subtypes. Hereby, the 5th percentile proved to be the most promising parameter. Graphical depiction of these values can be used as a heatmap with highlighted areas of low ADC values indicating malignant tumor tissue which might be beneficial for biopsy and surgical management. The comparison and subtraction of ADC heatmaps prior to and after chemotherapy could be useful for therapy response assessment of the whole tumor tissue and for planning of further localized interventions in areas of low response.

## Supplementary Information

**Additional file 1.** Bland-Altman analysis of tumor volumetry. Additional file 1 displays the Bland-Altman analysis of semi-automated diffusion weighted imaging tumor volumetry compared to manual standard segmentation (T1 and 2 weighted imaging). Volume is given in ml.

## Data Availability

The datasets used and/or analyzed during the current study are available from the corresponding author on reasonable request.

## Abbreviations

NB: Neuroblastoma
GNB: Ganglioneuroblastoma
GN: Ganglioneuroma
INRG: International Neuroblastoma Risk Group
US: Ultrasound
CT: Computed tomography
MRI: Magnetic resonance imaging
123I-MIBG: 123Iodine-meta-iodobenzylguanidine
DWI: Diffusion weighted imaging
ADC: Apparent diffusion coefficient
ROI: Region of interest
GTR: Gross total resection
LR: Low risk
IR: Intermediate risk
HR: High risk
VIBE: Volumetric interpolated breath-hold examination
ROC: Receiver operating characteristics
PET: Positron emission tomography
